# Moral reasoning, moral decision‐making, and empathy in Korsakoff’s syndrome

**DOI:** 10.1111/jnp.12233

**Published:** 2020-11-27

**Authors:** Erik Oudman, Sioux van Stigt Thans, Estrella R. Montoya, Albert Postma

**Affiliations:** ^1^ Experimental Psychology Helmholtz Institute Utrecht University Utrecht The Netherlands; ^2^ Lelie Care Group Slingedael Korsakoff Center Rotterdam The Netherlands

**Keywords:** executive functioning, Korsakoff syndrome, moral reasoning, social skills

## Abstract

Korsakoff’s syndrome (KS) is a neuropsychiatric disorder, caused by a vitamin B1 deficiency. Although it is known that patients with KS display diminished theory of mind functioning and frequently exhibit marked antisocial interactions little attention has so far focused on the integrity of moral decision‐making abilities, moral reasoning, and empathy. In an experimental cross‐sectional design, 20 patients diagnosed with KS, and twenty age‐, education‐, and gender‐equivalent healthy participants performed tests assessing moral decision‐making, moral reasoning maturity, empathy, and executive functioning. Participants were administered the Moral Behaviour Inventory (MBI) for everyday moral dilemmas, and ten cartoons of abstract moral dilemmas. Responses were scored according to the Kohlberg stages of moral reasoning. Empathy and executive functioning were assessed with the Interpersonal Reactivity Index (IRI) and the Frontal Assessment Battery (FAB). In contrast to frontal traumatic brain injury patients, KS patients did not display a utilitarian bias, suggesting preserved moral decision‐making abilities. Of interest, KS patients had significantly lower levels of moral reasoning maturity on everyday moral dilemmas, and abstract moral dilemmas. In patients, empathy was moderately related to the level of moral maturity on both tasks, while executive functioning was not. In conclusion, KS patients have preserved moral decision‐making abilities, but their moral reasoning abilities are poorer in everyday and abstract situations. Lower moral reasoning abilities and lower levels of empathy together may be responsible for adverse social functioning in KS.

## Background

Korsakoff’s syndrome (KS) is a neuropsychiatric disorder caused by vitamin B1 deficiency and concomitant alcoholism (Kopelman, Thomson, Guerrini, & Marshall, 2009). Patients with KS suffer from severe declarative memory disorder and often show impairments in executive functioning like inhibitory control, planning, and set‐shifting (Fama, Marsh, & Sullivan, 2004; Moerman et al., 2019). Neuropsychiatric symptoms are also highly prevalent in KS, such as irritability, aggression, and disinhibition of behaviour (Gerridzen et al., 2018).

Socially maladaptive functioning in KS is common. For instance, there is evidence that social interactions are minimal, and patients frequently experience a strong sense of social isolation and loneliness (Oudman, van Dam, Postma, 2018). Also, patients with KS and chronic alcohol use disorders are strongly represented in the criminal justice system (Coulton et al., 2012). Possible explanations for social dysfunction in KS can be found in loss of integrity of social skills, such as emotion recognition (Drost, Postma, & Oudman, 2018; Montagne, Kessels, Wester, & de Haan, 2006), theory of mind (Drost et al., 2018), social perspective taking (Drost et al., 2018; Oosterman, de Goede, Wester, van Zandvoort, & Kessels, 2011), and understanding faux pas situations (Drost et al., 2018).

A central component of social cognition in determining appropriate social decisions and behaviours is moral decision‐making. Moral decision‐making refers to any sort of decision – including judgments, evaluations, and response choices – made within the ‘moral domain’ (Smetana, 2006), that is, decisions regarding moral issues or principles, such as justice, harm fairness, and care. A moral decision can be a response decision about how to behave in a moral dilemma, a situation with moral rules and principles attached, where a response choice is required but difficult to make because of complex balance of pro’s and con’s (Garrigan, Adlam, Langdon, 2018). In typical moral dilemmas, the participant is required to perform one out of two actions (Greene, Sommerville, Nystrom, Darley, & Cohen, 2001). Both abstract and everyday moral dilemmas exist. A now famous example of an abstract moral dilemma requiring moral decision‐making is ‘the switch dilemma’ (Fischer & Ravizza, 1992). In this scenario, a loose trolley rolls down a track heading for five people. The five people will be killed by the force of the trolley unless the participant flips a switch to divert the trolley onto another track upon which only one person is situated. An example of an everyday moral dilemma is whether or not to sell someone a defective car.

Dealing with moral dilemmas has been the topic of investigation in many studies. According to Greene's (2001) dual‐process model of moral decision‐making, when faced with a moral question where one person must be hurt to aid others, reasoners intuitively detect that harming others is wrong, but have to engage in demanding deliberation to realize that harm can be acceptable depending on the consequences. The fast and emotional‐driven response (‘never use or harm a person as a means to an end’) is referred to as the deontological response, while the slow and deliberative response is referred to as the utilitarian response (‘maximizing the aggregate welfare’) (Conway & Gawronski, 2013; Greene, Nystrom, Engell, Darley, & Cohen, 2004; Greene et al., 2001). Recent neuroimaging evidence has characterized how emotional and cognitive processes are computed and interact. These studies show that emotional and utilitarian appraisals are independently encoded in anatomically segregated pathways before getting integrated in the ventromedial prefrontal cortex (Hutcherson, Montaser‐Kouhsari, Woodward, & Rangel, 2015; Shenhav & Greene, 2014).

Although the dual‐process theory of moral decision‐making is widely accepted, a shift to a more dynamic model of moral decision‐making has been advocated, to both understand how moral decisions are made, but also what processes are required to develop mature moral decision‐making skills; referred to as moral reasoning abilities (Van Bavel, FeldmanHall, Mende‐Siedlecki, 2015). Moral reasoning represents the mental process through which judgments about right and wrong and social conventions are made. In moral dilemmas, moral reasoning can be investigated by asking participants to give a justification for the moral decision they make (Beauchamp, Vera‐Estay, Morasse, Anderson, & Dooley, 2019). From a developmental point of view, moral reasoning is acquired in stages, with the highest stages representing a comparison of one's own actions with expectations of society and the lowest stages representing a punishment‐driven approach (Arsenio & Lemerise, 2004; Kohlberg, 1971). In the lowest level of moral reasoning, referred to as pre‐conventional moral reasoning, morality of actions is based on the direct consequences. In conventional moral reasoning, people make decisions conforming to social rules or laws of the legal system. In post‐conventional reasoning, individuals do not just conform to an existing set of rules, but make moral decisions based on their own values and beliefs. They realize that individual's own perspective may prevail over society's view (Kohlberg, 1971; Snarey & Samuelson, 2008).

Earlier studies in other clinical groups found that patients with frontal lesions or frontal Traumatic Brain Injury (TBI) show a preference for utilitarian responses (Caldwell et al., 2015; Greene, Morelli, Lowenberg, Nystrom, & Cohen, 2008; Koenigs et al., 2007; Kahane et al., 2011). In these studies, patients are more willing to judge moral violations as acceptable behaviour in dilemmas that require the harming or killing of persons to achieve goals, and do so more quickly (Greene et al., 2001; Martins, Faisca, Esteves, Muresan, & Reis, 2012; Rowley, Rogish, Alexander, & Riggs, 2018). Moreover, research in patients with alcohol use disorders (AUD) showed that moral dilemmas often provoked a utilitarian bias in this population (Khemiri, Guterstam, Franck, & Jayaram‐Lindström, 2012; Carmona‐Perera et al., 2012). When asked for justifications for moral decisions, adolescents with traumatic brain injury had poorer moral reasoning capacities than healthy control subjects of the same age (Dooley, Beauchamp, & Anderson, 2010; Beauchamp, Dooley, Anderson, 2013). Of interest, some studies did not find a utilitarian bias in moral decision‐making, but found that the justifications for the moral decisions represented a lower level of moral reasoning (Beauchamp et al., 2013; Beauchamp et al., 2019).

Importantly, there are currently no studies available on moral functioning in KS. Therefore, in the present study we wanted to elucidate moral decision‐making and moral reasoning abilities in patients with KS. We presented abstract and everyday moral dilemmas to twenty patients diagnosed with KS, and age‐ and education‐matched controls. Half of the abstract moral dilemmas were intuitively deontological, more often provoking a ‘no’ response. The other half are intuitively utilitarian, more often provoking a ‘yes’ response. We assessed both the responses to moral dilemmas as an assessment for moral decision‐making and the moral reasoning maturity behind moral decisions based on justifications. We also assessed empathy based on self‐reports. Empathy can be defined as the ability to imagine oneself in another’s place to understand and respond to other’s feelings, ideas, or emotions (Decety & Jackson, 2006). It has been closely linked to moral reasoning abilities (Decety & Cowell, 2015). We expected a utilitarian bias in KS patients compared to control subjects on everyday and abstract moral dilemmas, based on prior research in AUD and frontal lesion patients. Moreover, we also expected lower levels of moral reasoning and empathy, based on prior studies in young TBI patients for moral reasoning and results in AUD patients for empathy. Also, because moral reasoning is possibly related to executive functioning (Cottone, Drucker, Javier, 2007) and empathy (Decety & Cowell, 2015), we wanted to predict moral reasoning abilities in a linear regression model.

## Materials and methods

### Participants

Twenty patients (12 male) diagnosed with KS participated in this study. A summary of demographic variables is presented in Table 1. They were all inpatients of a long‐term care facility for KS patients and fulfilled the DSM‐V criteria for ‘Alcohol‐induced major neurocognitive disorder, Amnestic Confabulatory type’ (code: 291.1) (American psychiatric association, 2013) and the characteristics of KS described by Kopelman (Kopelman, 2002). The patients were in the chronic, amnestic stage of the Korsakoff Syndrome and not in a Wernicke psychosis. Exclusion criteria were illiteracy, presence of additional neurological disorders (traumatic brain injury, epilepsy, stroke, or brain tumour), acute psychiatric conditions (psychosis, major depression, etc.) and physical conditions interfering with the testing procedure. Twenty age‐, education‐, and gender‐matched healthy participants (12 male) were included as a reference group (See Table 1). The study was conducted according to the declaration of Helsinki and for all patients written informed consent was obtained.

**Table 1:** Demographic characteristics

	KS (*n* = 20)	HC (*n* = 20)	Statistics
Gender (m: f)^a^	12: 8	12: 8	χ^2^ (1, *N* = 40) = 0.00, *p* = 1.00
Age (*M*, *SD*)^b^	61.75 (7.83)	64.10 (9.44)	*t*(38) = −.856, *p = .397*
Education level (*M*, *SD*) ^c^	4.50 (1.24)	4.95 (0.95)	*t(38) = −1.294, p = .203*

^a^ Gender ratio male: female.

^b^ Age in years.

^c^ Educational level was assessed in seven categories: one, primary school; seven, academic degree (Verhage, 1964).

### Materials

#### Abstract moral dilemmas – Moral decision‐making

Ten abstract moral dilemmas, earlier developed by Kahane et al. (2011), were presented with a comparable administration method as Rowley et al. (2018). The dilemmas were presented as cartoon drawings accompanied with short stories. Five dilemmas were Intuitively Utilitarian, more frequently provoking a ‘yes’ response in earlier research, while five others were Intuitively Deontological, more frequently provoking a ‘no’ response.

The experimenter read the dilemmas aloud once, before inviting participants to make a judgment on what they should do. The dilemmas were read aloud to ensure better story comprehension. Answers to each dilemma were recorded and subsequently categorized (Intuitively Utilitarian/Intuitively Deontological/yes, no).

#### Abstract moral dilemmas – Moral reasoning

Based on the So‐Moral task developed by Beauchamp, Dooley, and Anderson (2013), participants were also asked to provide a justification for the choice they made for the ten abstract moral dilemmas (Rowley, Roghish, Alexander, and Riggs, 2018). All answers were written down, and subsequently scored using the moral maturity coding scheme based on Beauchamp et al., (2013). Per dilemma, a minimum score of one points, and a maximum score of three points could be scored. A score of one point was given for a pre‐conventional style of reasoning, a score of two points was given for a conventional line of reasoning, and three points were given for a post‐conventional line of reasoning. Classic examples of pre‐conventional moral reasoning styles are: ‘I will do this, because it will make me better’ or ‘If I do not receive punishment, I will do it’. Examples of conventional moral reasoning styles are: ‘Others expect me to do so’ or ‘I’m not allowed to do this, because of the law’. A post‐conventional reasoning example is: ‘There are laws that do tell me (not) to do so, but because the outcome for one person is very detrimental, it is ok to break the law’.

Moreover, the average level of reasoning was calculated for the five Intuitively Deontological items, and the five Intuitively Utilitarian items.

#### Moral behaviour inventory – Moral decision‐making

The Moral Behaviour Inventory (MBI) is a 24‐item questionnaire developed by Mendez, Anderson, and Shapira (2005) which consists of short everyday dilemmas. This questionnaire was applied to evaluate the participants’ knowledge of everyday social and moral norms in addition to the moral reasoning skills. Examples of the items are ‘Take the last seat on a crowded bus’’ or ‘’Always let others pay at a restaurant’. Items 4, 9, 20, and 21 were not included based on inappropriateness for a KS patient group. Item 4, item 20 and item 21 refer to situations that are not commonly occurring in the lives of the patients (driving a car, doing homework or having duty as a jury member). Item 9 refers to a situation that KS patients might find offensive, because they sometimes have been homeless (driving out homeless people from the community). The experimenter read the dilemmas aloud once, before inviting participants to make a judgment on what they should do. The dilemmas were read aloud to ensure better story comprehension. Answers to each dilemma were recorded and subsequently categorized (yes, no).

#### Moral behaviour inventory – Moral reasoning

Based on the So‐Moral task (Beauchamp, Dooley, Anderson, 2013), participants were asked to provide a justification for the choice they made for the MBI dilemmas (Mendez, Anderson, Shapira, 2005). All answers were written down, and subsequently scored using the moral maturity coding scheme based on Beauchamp et al. (2013). Per dilemma, a minimum score of one point, and a maximum score of three points could be scored. A score of one point was given for a pre‐conventional style of reasoning, a score of two points was given for a conventional line of reasoning, and three points were given for a post‐conventional line of reasoning. The average level of reasoning was calculated for all 20 items.

#### Empathy – Interpersonal reactivity index

To index empathy, two subscales ‘empathic concern’ (EC) and ‘perspective‐taking’ (PT) of the Interpersonal reactivity index (IRI) were used (De Corte et al., 2007; Davis, 1980). The two subscales each consists of seven items. For each item the participants had to indicate how well it describes them on a 5‐point Likert scale ranging from 0 (*does not describe me well*) to 4 (*describes me very well*). Scale scores were computed by summing up the scores for each scale, which can result in a score from 0 (minimum) to 28 (maximum).

#### Executive Functioning ‐ Frontal Assessment Battery

The Frontal Assessment Battery (FAB) is a short screening test which was used to assess a background variable, frontal lobe function. The FAB consists of six subtests: conceptualization, mental flexibility, motor programming, and resistance to interference, inhibitory control, and environmental autonomy. Subtest scores can range from 0 (minimum) to 3 (maximum). With the global score of these subtests, dysexecutive functioning can be evaluated (Dubois, Slachevsky, Litvan, & Pillon, 2000).

### Data analysis

All test scores were compared between KS patients and healthy controls. For abstract moral dilemmas, moral decision‐making data (yes/no responses) were transformed into proportions for each participant. T‐tests were performed on the average utilitarian proportion for the five abstract intuitively utilitarian dilemmas (sum of categorized responses/5) and for the five abstract intuitively deontological items (sum of categorized responses/5). Independent samples *t*‐test was used to compare scores on abstract moral dilemmas, the MBI, the empathy ‘interpersonal reactivity index’, the empathy ‘perspective taking’, and the FAB. To index moral reasoning, the justifications for intuitively deontological dilemmas, intuitively utilitarian dilemmas, and everyday moral dilemmas (MBI) were compared using an independent *t‐*test. Due to violation of the assumption of normality and the assumption of homogeneity of variances for the total FAB scores in the healthy control group, the non‐parametric Mann–Whitney *U* test was applied. We included three linear regressions to predict moral reasoning on intuitively utilitarian (abstract), intuitively deontological (abstract), and everyday moral dilemmas based on the levels of empathy (empathic concern and perspective taking subtest of the IRI), and executive functioning (FAB), with level of education, age, and group as control variables.

## Results

All participants completed the entire study protocol. There were no statistically significant group differences regarding age, sex, and education of the participants (see Table 1).

### Abstract moral decision‐making

**Figure 1:**
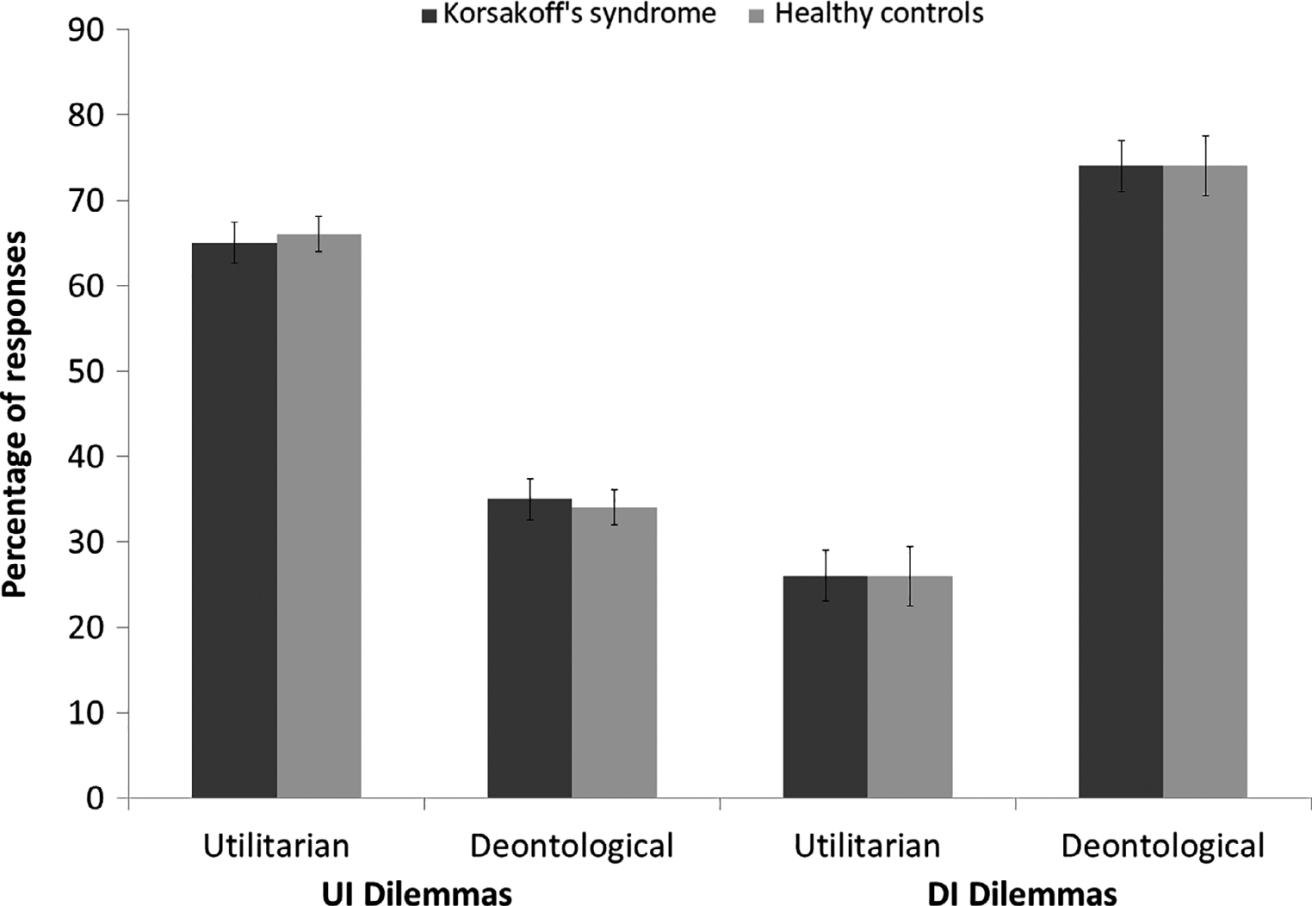
Judgement for Korsakoff’s syndrome patients (*n* = 20) and matched controls (*n* = 20). Average percentage of utilitarian and deontological responses in the Korsakoff’s syndrome group (*n* = 20) and control group (*n* = 20), in UI and DI dilemmas. Error bars are standard error of the mean.

In KS patients, the proportion of intuitive judgements was significantly higher than the 0.5 baseline chance level in both UI (‘yes’ items), *t*(19) = 3.135, *p* < .01, 95% CI (0.050, 0.250), and DI (‘no’ items), *t*(19) = 4.000, *p* < .01, 95% CI (0.114, 0.366) dilemmas (see Figure 1, left). The patients showed a significant preference for deontological judgements (also seen as ‘no responses’ to moral dilemmas) when all dilemmas were pooled together and compared against a 0.5 baseline *t*(19) = 4.333, *p* < .01, 95% CI (0.101, 0.289).

In control subjects, the proportion of intuitive judgements was significantly higher than the 0.5 baseline in both Intuitively Utilitarian, *t*(19) = 3.875, *p* < .01, 95% CI (0.074, 0.246), and Intuitively Deontological, *t*(19) = 3.442, *p* < .01, 95% CI (0.094, 0.386), dilemmas (see Figure 1, right). The control group showed a significant preference for deontological judgements (‘no responses’) when all dilemmas were pooled together and compared against a 0.5 baseline *t*(19) = 5.119, *p* < .01, 95% CI (0.118, 0.282).

Both patients and control group did show comparable responses on the utilitarian proportion for the average of the five intuitively utilitarian abstract moral dilemmas, *t*(38) = −.158, *p* = .88, 95% CI (−0.138, 0.118), δ = 0.050. Also, on the intuitively deontological moral dilemmas the decisions were comparable in patients and controls, *t*(38) = 0.0, *p* = .15 95% CI (−0.190, 0.030), δ = 0.

### Abstract moral reasoning

Of importance, patients showed lower levels of moral reasoning on intuitively utilitarian abstract moral dilemmas, *t*(38) = −4.835, *p* < .01, 95% CI (−0.993, −.406), δ = 1.529. In Figure 1, the number of participants in the different stages of moral development is represented for intuitively utilitarian moral dilemmas.

**Figure 2:**
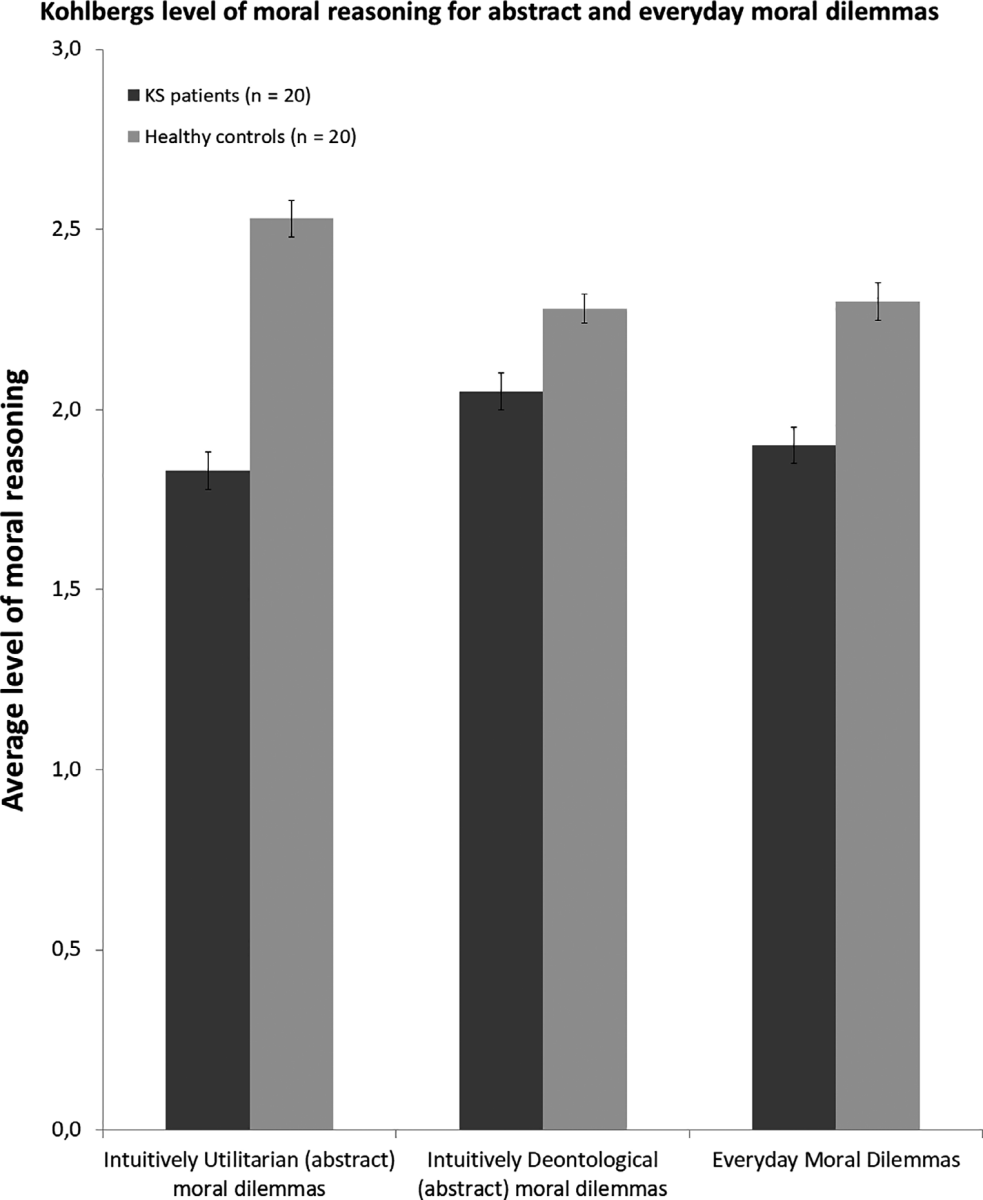
Average level of moral reasoning with one representing the pre‐conventional level, two the conventional and three the post‐conventional level. Bars represent the average level of moral reasoning for intuitively utilitarian moral dilemmas (left), intuitively deontological moral dilemmas (middle), and everyday moral dilemmas (right) for the Korsakoff’s syndrome patients (*N* = 20) and the healthy participants (*N* = 20) based 10 intuitively utilitarian moral dilemmas. Error bars represent a standard error of the mean.

On intuitively deontological moral dilemmas, patients and healthy subject had comparable levels of moral reasoning, *t*(38) = −1.769, *p* = .09, 95% CI (−0.493, −.033), δ = 0.559. In Figure 2, average level of moral reasoning was represented for all moral dilemmas.

### Everyday moral decision‐making and moral reasoning – Moral behaviour inventory

Both KS patients and healthy controls had comparable MBI scores, *t*(38) = −.752, *p* = .46, 95% CI (−5.901, 2.705), δ = 0.238. Subsequently, the lines of reasoning were scored according to the Kohlberg model. KS patients scored lower in comparison to the HC group, *t*(38) = −2.76, *p* < .01, 95% CI (−.694, −.106), δ = 0.872.

### Empathy

There were no significant differences between KS patients and the HC group on the ‘empathic concern’ (EC) subscale, *t*(38) = –1.358, *p* = .18, 95% CI (−5.853, 1.153), δ = 0.430, and the ‘perspective‐taking’ subscale, *t*(38) = –1.502, *p* = .14, 95% CI (−6.105, 0.905), δ = 0.475, suggesting no differences between the self‐reported levels of empathy.

### Executive dysfunctioning

The FAB score was statistically different between patients and controls, *t*(38) = 2.68, *p* < .05, δ = 0.846, suggesting lower levels of executive functioning in the KS patients compared to the healthy subjects.

### Executive functioning, empathy, and moral reasoning skills

**Figure 3:**
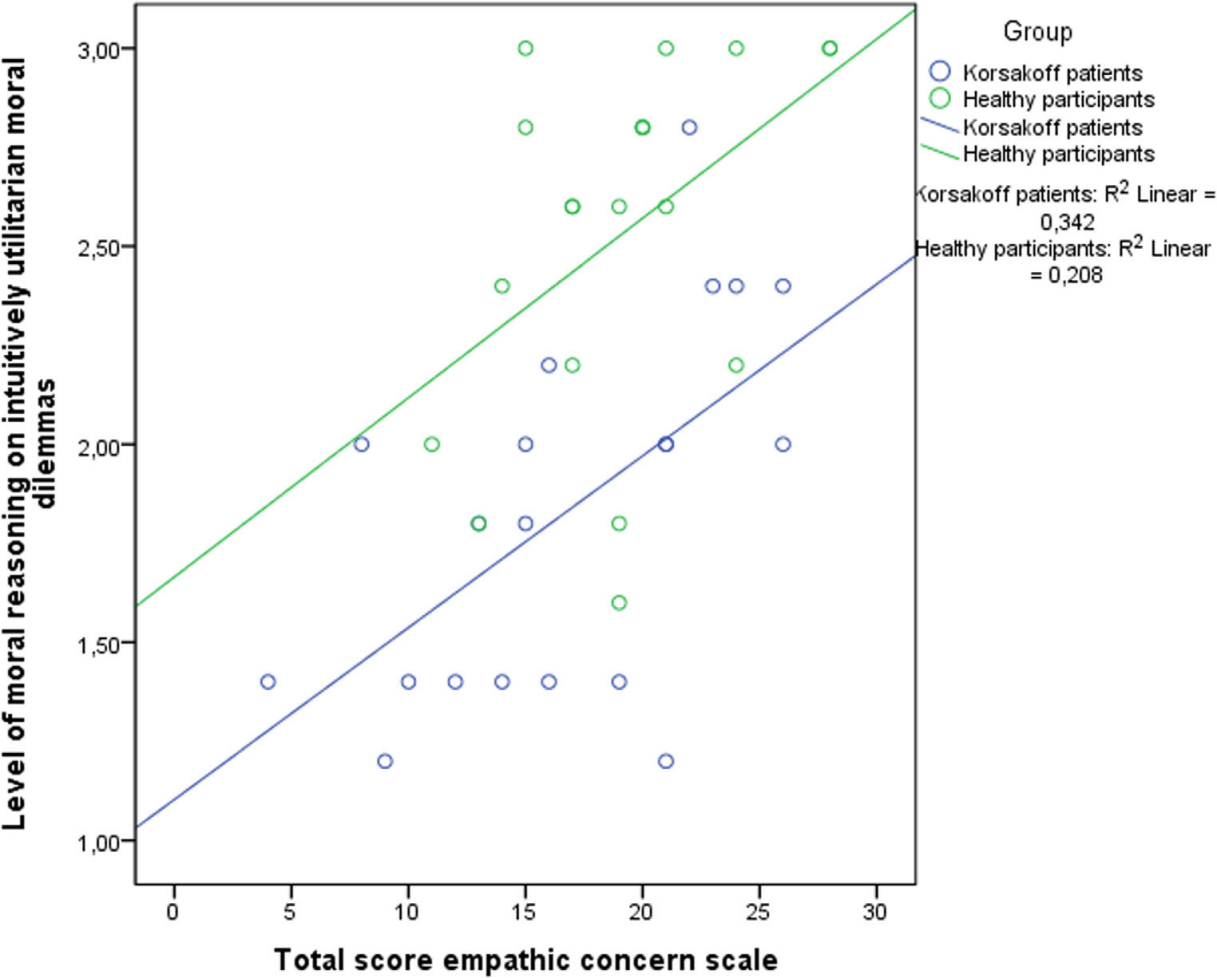
Scatterplot with regression lines showing the relationship between the score on the Empathic Concern scale of the Interpersonal Reactivity Index and level of moral reasoning on intuitively utilitarian abstract moral dilemmas for Korsakoff’s syndrome patients (*N* = 20) and healthy participants (*N* = 20).

A multiple linear regression was performed to determine whether the level of education, age, gender, and group of participants as control variables, and the empathic concern scale of the IRI, the perspective taking scale of the IRI and the FAB as predictors, predicted the level of moral reasoning on intuitively utilitarian abstract moral dilemma. Casewise diagnostics did not detect any influential cases. Using the enter method, it was found that the model including all predictors and control variables explained a significant amount of the variance in the level of moral reasoning on intuitively utilitarian abstract moral dilemma, *F*(7,32) = 7.667, *p* < .01, *R* ^2^ = .62, *R* ^2adjusted^ = .545. The analysis shows that the level of moral reasoning on intuitively utilitarian abstract moral dilemma was not significantly predicted by level of education, β = .1 *t*(39) = 1.43, *p* = .16; age, β = −.01 *t*(39) = −1.18, *p* = .25; gender (0 for male, 1 female), β = −.16 *t*(39) = −1.17, *p* = .25; total score on the perspective taking scale, β = .00 *t*(39) = .24, *p* = .81, and the FAB, β = −.01 *t*(39) = −.36, *p* = .72. The level of moral reasoning on intuitively utilitarian abstract moral dilemma was significantly predicted by group (0 for Korsakoff’s syndrome, 1 for controls), β = .60 *t*(39) = 4.07, *p* < .01, and total score on the empathic concern scale, β = .04 *t*(39) = 2.82, *p* < .01. The relationship between the empathic concern scale and the level of moral reasoning on intuitively utilitarian abstract moral dilemmas is depicted in Figure 3.

A second multiple linear regression was performed to determine whether the level of education, age, gender, and group of participants as control variables, and the empathic concern scale of the IRI, the perspective taking scale of the IRI and the FAB as predictors, predicted the level of moral reasoning on intuitively deontological abstract moral dilemma. Casewise diagnostics did not detect any influential cases. Using the enter method, it was found that the model including all predictors and control variables did not explain a significant amount of the variance in the level of moral reasoning on intuitively deontological abstract moral dilemmas, *F*(7,32) = 1.889, *p* = .10, *R* ^2^ = .294, *R* ^2adjusted^ = .139. None of the control variables or predictors were significant (*p*s > .05).

A third multiple linear regression was performed to determine whether the level of education, age, gender, and group of participants as control variables, and the empathic concern scale of the IRI, the perspective taking scale of the IRI and the FAB as predictors, predicted the level of moral reasoning on everyday moral dilemma. Casewise diagnostics did not detect any influential cases. Using the enter method it was found that the model including all predictors and control variables did explain a significant amount of the variance in the level of moral reasoning on everyday moral dilemmas, *F*(7,32) = 3.121, *p* = .01, *R* ^2^ = .406, *R* ^2adjusted^ = .276. The analysis shows that the level of moral reasoning on everyday moral dilemmas was not significantly predicted by level of education, β = −.1 *t*(39) = −1.240, *p* = .22; age, β = –.01 *t*(39) = −1.235, *p* = .23; gender (0 for male, 1 female), β = −.21 *t*(39) = −1.45, *p* = .16; total score on the empathic concern scale, β = .02 *t*(39) = 1.10, *p* = .28; total score on the perspective taking scale, β = .02 *t*(39) = 1.16, *p* = .26, and the FAB, β = .027 *t*(39) = .63, *p* = .53. Group (0 for Korsakoff’s syndrome, 1 for controls) did show a trend towards predicting the level of moral reasoning on everyday moral dilemmas, β = .33 *t*(39) = 2.02, *p* = .05.

## Discussion

The aim of this study was to investigate moral reasoning and moral decision‐making abilities in KS patients. We expected a utilitarian bias in KS patients on everyday and abstract moral dilemmas. Moreover, we also expected lower levels of moral reasoning and empathy. Our results indicated an absence of a utilitarian bias and relatively intact empathy. Of importance, KS patients had lower levels of moral reasoning maturity compared to matched individuals.

In light of the dual‐process model of moral decision‐making (Greene, 2001), the present findings do not support this theory, because KS patients make comparable moral decisions as healthy subjects. KS patients to make more utilitarian judgments than controls. Importantly, KS patients did not show a stronger utilitarian bias on moral dilemmas, in contrast to earlier research on frontal lesion patients, TBI patients, and chronic AUD patients (Bartels & Pizarro, 2011; Carmona‐Perera et al., 2014; Caldwell et al., 2015; Greene et al., 2008; Kahane et al., 2011; Khemiri et al., 2012; Koenigs et al., 2007). Earlier research in psychopaths indicated that there is a discrepancy between understanding the distinction between right and wrong, and caring about the knowledge and consequences of making a moral decision (Cima, Tonnaer, & Hauser, 2010). Also patients diagnosed with KS might understand the distinction between right and wrong, but only care for direct positive or negative outcomes for themselves irrespective of the law. Patients with KS developed their condition later in life, having a relatively uncomplicated development, leading to relatively intact moral knowledge in comparison with early TBI patients (Beauchamp et al., 2019; Kahane et al., 2011).

Interestingly, also Beauchamp et al. (2013) found no utilitarian bias but reduced levels of moral maturity in adolescents with TBI, relating to adverse social outcome. In line with Beauchanp et al. (2013) in the present study KS patients showed lower levels of moral maturity compared to matched controls. Moral reasoning was more often based on ‘pre‐conventional’, punishment or reward driven reasoning, than on ‘conventional’ law‐based reasoning. Earlier research in TBI suggested a comparable decline in moral reasoning abilities in this population (Beauchamp et al., 2013; Martins et al., 2012).

In everyday moral dilemmas and relatively everyday intuitively utilitarian moral dilemmas, KS patients more frequently tended to display a punishment‐ or direct gain‐driven approach (‘I will do this, because it will make me better/I do not receive punishment’) than healthy controls, while in extreme moral dilemmas patients and controls displayed more conventional moral reasoning (‘I'm not allowed to do this, because of the law’). This pattern was not visible in making the actual moral decisions, with all categories resulting in a comparable yes/no‐tendency between KS patients and controls. Also in the study by Beauchamp et al. (2019) moral reasoning was compromised in the severe and moderate TBI groups with intact moral decision‐making and lower levels of moral reasoning, while in the mild TBI group moral decisions were more intuitively utilitarian and moral reasoning was of a lower level. On the level of decisions, no differences were found between KS and HCs, possibly because the different reasoning patterns that these groups display could lead to the same decision. For example, deontological decisions could be driven by self‐oriented emotions such as punishment and reward in KS patients and by other‐oriented emotions such as empathy in HCs.

Several studies have indicated that high levels of moral reasoning are associated with intact empathy (Hoffman, 2001) and executive functioning (Cottone, Drucker, & Javier, 2007). The impairments in executive and emotional functioning often experienced in KS combined with their history of criminal behaviour (Maharasingam, Macniven, & Mason, 2013; Oscar‐Berman, 2012) led to the expectation that lower levels of moral reasoning would relate to lower levels of empathy in patients. Surprisingly, the patients and controls did not differ in their level of empathy, whereas in previous research patients with KS do show emotional abnormalities like lack of empathy (Oscar‐Berman, 2012). We like to mention here that a more suitable technique could be to use a psychophysical measurement of empathy such as, the Multifaceted Empathy Test or the Movie for the Assessment of Social Cognition, rather than a self‐report measure (Dziobek et al., 2006; Dziobek et al., 2008). Even though no differences in empathy levels were found between the groups, the current study showed a positive relationship between the empathic concern scale of the IRI and moral reasoning on intuitively utilitarian moral dilemmas. This suggests that the patients with standard levels of empathy are more sensitive for personal consequences of moral decisions, leading to more punishment‐driven motivation for their moral decisions. In earlier research, empathic concern (i.e., feelings of warmth and compassion in response to someone in distress) was found to be significantly related to more deontological responses (Gleichgerrcht & Young, 2013).

In this study, levels of executive functioning were not related to moral reasoning, despite a group difference between patients and controls. Earlier research also found no relationship between theory of mind and executive functioning in KS (Drost et al., 2018). It is possible that a more elaborate and longer test battery for executive functioning would have found some relationship with moral reasoning. In healthy subjects, particularly higher levels of moral reasoning related robustly with post‐conventional reasoning (Cottone et al., 2007). Because we did not find individual effects of the FAB on intuitively deontological or intuitively utilitarian moral dilemmas, we did not investigate executive functioning as a possible explanation the dissociation between both types of dilemmas. In a larger study, it would be helpful to investigate the relationship between executive functioning and multiple sets of moral reasoning with an instrument assessing subcomponents of executive functioning.

This study has a number of limitations. In future research, it would be relevant to apply real‐life testing procedures or virtual reality testing procedures, specifically because the validity of self‐report vignettes is limited and laboratory‐based testing could include additional physiological measures (Bostyn, Sevenhant, & Roets, 2018; Francis et al., 2016; Patil, Cogoni, Zangrando, Chittaro, & Silani, 2014). It would also be relevant to test whether decisions are based on negative consequences for the participant, by comparing situations that are beneficial for the participant, have neutral consequences, and are not beneficial for the participant. This would be particularly relevant, because the present study found evidence for altered moral reasoning with intact moral decision‐making. It could be that testing different types of decisions would result in more clear‐cut difficulties in KS.

In conclusion, results of this study indicate that KS patients do respond as healthy controls to moral dilemmas, but exhibit moral reasoning abilities on a markedly lower level, potentially putting them at risk for unthoughtful social decision‐making and maladaptive behaviour.

## Authors contributions

Erik Oudman (Conceptualization; Data curation; Formal analysis; Investigation; Methodology; Project administration; Resources; Software; Supervision; Validation; Visualization; Writing – original draft; Writing – review & editing) Sioux van Stigt Thans (Conceptualization; Data curation; Investigation; Methodology; Visualization; Writing – original draft) Estrella R. Montoya (Conceptualization; Investigation; Methodology; Writing – original draft) Albert Postma (Conceptualization; Supervision; Validation; Writing – original draft; Writing – review & editing).

## Data availability statements

The data that support the findings of this study are available on request from the corresponding author. The data are not publicly available due to privacy or ethical restrictions.
